# Comparison of Patient-Reported Outcome from Neck-Preserving, Short-Stem Arthroplasty and Resurfacing Arthroplasty in Younger Osteoarthritis Patients

**DOI:** 10.1155/2015/817689

**Published:** 2015-05-26

**Authors:** Marius Dettmer, Amir Pourmoghaddam, Stefan W. Kreuzer

**Affiliations:** Memorial Bone & Joint Research Foundation, 1140 Business Center Drive No. 101, Houston, TX 77043, USA

## Abstract

Hip resurfacing has been considered a good treatment option for younger, active osteoarthritis patients. However, there are several identified issues concerning risk for neck fractures and issues related to current metal-on-metal implant designs. Neck-preserving short-stem implants have been discussed as a potential alternative, but it is yet unclear which method is better suited for younger adults. We compared hip disability and osteoarthritis outcome scores (HOOS) from a young group of patients (*n* = 52, age 48.9 ± 6.1 years) who had received hip resurfacing (HR) with a cohort of patients (*n* = 73, age 48.2 ± 6.6 years) who had received neck-preserving, short-stem implant total hip arthroplasty (THA). Additionally, durations for both types of surgery were compared. HOOS improved significantly preoperatively to last followup (>1 year) in both groups (*p* < 0.0001, *η* ^2^ = 0.69); there were no group effects or interactions. Surgery duration was significantly longer for resurfacing (104.4 min ± 17.8) than MiniHip surgery (62.5 min ± 14.8), *U* = 85.0, *p* < 0.0001, *η* ^2^ = 0.56. The neck-preserving short-stem approach may be preferable to resurfacing due to the less challenging surgery, similar outcome, and controversy regarding resurfacing implant designs.

## 1. Introduction

Osteoarthritis (OA) is a degenerative disease that causes damage to joint structures and cartilage tissue. Although some nonsurgical treatments exist, often, an intervention in the form of arthroplasty becomes necessary. In the case of hip OA, the standard approach for patients undergoing surgical treatment in the USA is total hip arthroplasty (THA), which is often based on porous-coated stems implanted into the femur and a cup counterpart (acetabulum) implanted into the hip socket [1]. Over a million arthroplasty surgeries are performed each year, and it is expected that this number will double in the coming decades [2]. The technique has been shown to be associated with positive clinical outcome [3–5]. However, it has also been noted that potential disadvantages of the standard implant procedure are proximal stress shielding [6] due to implant architecture and associated mechanical loading characteristics, which may affect bone metabolism and ultimately decrease implant stability. The success of a THA approach depends on good metaphyseal/diaphyseal (depending on porous-coated/non-porous-coated design) attachment of the femoral component for promotion of bone remodeling. In addition, there may be development of thigh pain in distal-coated stem approaches. Due to a lack of bone preservation associated with the intervention, potential future revisions may be difficult to perform and are more challenging.

**Figure 1:**
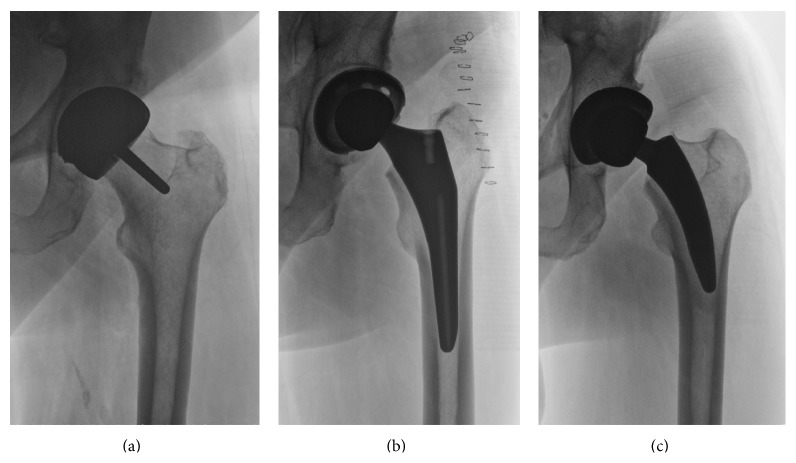
(a) HR implant, (b) traditional implant, and (c) neck-preserving, short-stem implant (Corin MiniHip). Most bone tissue is retained with HR (femoral head and neck, no significant intrusion of the femoral canal), whereas parts of the femoral neck are also preserved with the MiniHip approach.

For a number of specific patients, a hip resurfacing (HR) intervention may be an appropriate alternative to a total hip replacement. This approach includes the placement of a metal cap over the head of the femur and implantation of an artificial acetabulum (Figure 1(a)). Candidates for this intervention are mostly younger adults with relatively good bone quality [7–9], and the treatment may be appropriate for obese as well as nonobese patients [10]. Resurfacing implants preserve more bone tissue [11], which may have a number of advantages [12]. Overall positive functional and activity level outcomes have been reported in the short term and medium term [7, 13–17]. Furthermore, a relatively larger femoral head size may decrease the likelihood of dislocations [18, 19]. Previous investigations indicated that active individuals might further benefit from the use of this technique when it is combined with adequate rehabilitation. This may be a result of maintenance of anatomical characteristics, more normal gait kinematics, proprioception, and more optimal muscle activation characteristics after HR surgery [18]. However, as with alternative procedures, there have been reports of a number of disadvantages. There is a higher risk of femoral neck fractures and higher difficulty level of the surgery. Higher blood-ion levels due to the metal-on-metal design of specific implants and related implant wear have been reported [20–22], which has raised concerns in the medical community. Potential future of this treatment method is still being discussed [23, 24], but recent findings regarding toxicity of released nanoparticles and adverse reactions have been alarming [25, 26]. Additionally, there may be higher risk of earlier revisions, heterotopic ossification, and aseptic loosening [27]. Total hip arthroplasty in combination with new implant designs may lead to good results in younger patients [9] and minimize the reported concerns associated with current HR devices.

One alternative to THA using long-stem designs or resurfacing may be short-stem implants of less than 120 mm in length [28]. A characteristic of short-stem implants is a more proximal distribution of forces, which means they are dependent on appropriate metaphyseal fixation [29, 30]. Some designs, unlike traditional implants (Figure 1(b)), partially retain the femoral neck (Figure 1(c)), which is associated with biomechanical advantages [12] and higher stability [31, 32]. Femoral offset usually can be reproduced similarly to traditional implants [33], and several studies have reported good mid-term follow-up results [34–36]. Still, there is no consensus regarding potential, benefits, and limitations of this specific implant design. Potential issues are revision rates [37] and complications (e.g., periprosthetic fractures, subsidence) in some short-stem designs [6]. The surgical approach may be more challenging [38] and there are remaining questions, related to the optimal amount of porous coating [29]. Further, it is still debated whether there are specific patient groups that may be best suited for a short-stem, neck-preserving approach. For example, younger adults with higher activity levels and good bone quality may be better candidates for the intervention.

However, due to a lack of consensus in the literature regarding potential benefits or limitations/risks associated with each specific implant design, there is uncertainty regarding standardized recommendations towards one treatment over another. We designed a cohort study to compare outcome from two different interventions, which were MiniHip arthroplasty (Corin Group PLC, Cirencester, UK) and HR using a Cormet (Corin Group PLC, Cirencester, UK) implant, to evaluate which approach may be most favorable considering a sample of younger patients (≤55 years). We hypothesized that patients who were treated by resurfacing versus those having received MiniHip would demonstrate similar clinical results as assessed by patient-reported outcome.

## 2. Methods and Materials

This study was based on an IRB approved data registry (IRB # HSC-GEN-09-0143). All participants signed an informed consent form prior to data collection. HR or MiniHip surgery was indicated by a variety of factors, such as a relatively active lifestyle, less than 56 years of age for females, less than 65 years of age for males, adequate bone quality, and bone stock on acetabular component [39]. The surgeon has shifted to MiniHip treatment in cases where either of the two treatments was considered appropriate; therefore most HR surgeries were performed at an earlier time (June 2006–June 2011 for HR, August 2010 to April 2014 for MiniHip). Contraindications included large and multiple cysts, documented sensitivity to metal, or kidney issues.

The direct anterior approach used for HR is explained in detail elsewhere [35]. The MiniHip implant is based on a cementless, bicoated short-stem design aiming at bone conservation and proximal loading. The surgical approach included a presurgery templating routine (with determination of the center of the femoral head in line with the greater trochanter (based on X-rays)). During preoperative templating, the required implant size was evaluated which then dictated the required femoral neck resection level.

MiniHip surgery was also based on a minimally invasive anterior approach, using the ASIS and greater trochanter as reference points for incision. All cases were performed using a Stryker computer navigation system (Stryker Corp., Kalamazoo, MI). The incision was started about two centimeters distally and approximately 4–6 cm laterally to the ASIS. An oblique incision was made lateral to the space between the lateral side tensor fascia lata and medial side sartorius. The fascia covering the tensor fascia lata was then incised parallel to the muscle fibers. Acetabular preparation was based on preoperative planning and included implanting a Corin Trinity Advanced Bearing Acetabular System. On the femoral side, muscular dissection and femoral exposure were conducted, followed by the neck resection (including partial preservation of femoral neck). This step was followed by use of a curette to prepare the insertion of an awl for further bone preparation. Broaching with increasing sizes towards the templated broach size was performed, whereas the definite size broach was ensured to make three-point contact. The surgeon confirmed a stable fit, as determined by a good fit of the broach that filled the proximal femoral compartment, while sitting flush with the resected femoral neck. A trial neck and a trial head were then attached to evaluate appropriate sizes. After this, the implant was inserted and set in place in the femoral canal using an impactor. Range of motion, hip stability, and limb length were then checked and capsule, fascia, and skin were closed.

At study start, a total of 114 patients who previously had been diagnosed with osteoarthritis had undergone HR arthroplasty, and 436 patients had undergone MiniHip surgery. Both groups ASA classification ranged from I to III. In order to prevent a selection bias (in general, resurfacing patients were younger than MiniHip patients), we age-matched patients by limiting the maximum age to 55 years. This effectively eliminated significant age differences between groups. Only patients who had provided both presurgery and postsurgery survey data were included. The total number of patients included after matching was 125 (Table 1). Duration of surgery was recorded for each operation (where available) for later analysis.

Before surgery, patient survey data was obtained to compute a hip disability and osteoarthritis outcome score (HOOS). Additional patient surveys were conducted in the follow-up period after surgery (only patients having submitted both presurgery and postsurgery survey data were included in the study). For the purpose of comparison of the two methods, the most recent available follow-up information from each patient was included in the data analysis. Participants provided survey information electronically via an online portal. Surgery time and length of follow-up period were compared for both methods using Mann-Whitney tests.

Statistical analysis of HOOS consisted of a mixed-model MANCOVA approach with several dependent variables and follow-up period as covariate. Main outcomes were the subscores of the HOOS, that is, Symptoms, Pain, Activities of Daily Living (ADL), Sport/Recreation, and Hip-Related Quality of Life (QOL). There was one between-subjects factor, that is, surgery method (resurfacing versus MiniHip), and one repeated, within-subjects factor (i.e., presurgery versus postsurgery). Also, we analyzed potential interaction effects (pre- versus postsurgery method). Additionally, the analysis included the factor follow-up period as covariate. Bonferroni corrections were applied for multiple comparisons. Significance levels were set to *p* = 0.05. Effect sizes (partial-eta squared, *η* ^2^) were computed and are reported for each significant result.

## 3. Results

**Figure 2:**
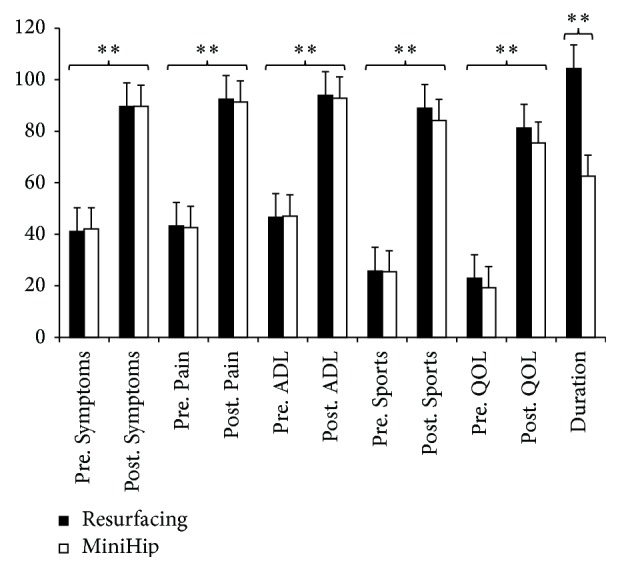
Comparison of presurgery and postsurgery HOOS (scale of 0–100) means (and standard error) and duration of surgery (in minutes) in MiniHip and resurfacing arthroplasty. Independent of surgery type, all subscale scores improved after surgery. The only group differences were found in comparison of surgery duration. ^*∗∗*^ *p* < 0.0001.

There was no significant main effect of group (MiniHip versus resurfacing). Statistical analysis showed a significant main effect of time (presurgery versus postsurgery), *F*(5,115) = 52.254, *p* < 0.0001, and *η* ^2^ = 0.69, but no interaction effects. Subsequent univariate analysis indicated improvement in all HOOS subscales (Figure 2), for Symptoms, *F*(1,119) = 174.748, *p* < 0.0001, and *η* ^2^ = 0.60; Pain, *F*(1,119) = 227.309, *p* < 0.0001, and *η* ^2^ = 0.66; ADL, *F*(1,119) = 212.029, *p* < 0.0001, and *η* ^2^ = 0.64; Sport, *F*(1,119) = 210.354, *p* < 0.0001, and *η* ^2^ = 0.64; QOL, *F*(1,119) = 162.471, *p* < 0.0001, and *η* ^2^ = 0.58. There were no significant interaction effects; method of surgery did not significantly alter outcome. Follow-up period differed significantly between MiniHip (495 days ± 281) and resurfacing group (1422 days ± 739), *U* = 623.0, *p* < 0.0001, but there was no significant effect of this covariate (outcome did not differ when controlled for follow-up period length). Results indicated that all subscores of the HOOS improved following surgery, independent of applied surgery method. Data for duration of the surgery was available from 34 patients in the resurfacing group and 70 patients in the MiniHip group. Mann-Whitney tests showed that resurfacing required significantly longer surgery times (104.4 min ± 17.8) than MiniHip surgery (62.5 min ± 14.8), *U* = 85.0, *p* < 0.0001, and *η* ^2^ = 0.56. There were three revisions in the resurfacing group (5.8%), due to fracture of the femoral neck. There were two revisions in the MiniHip group (2.7%), including one related to infection and one due to aseptic loosening. In an additional case there was a calcar fracture that was repaired with surgery implanting a 1.6 mm cable.

## 4. Discussion

THA has become significantly more common, especially in younger adults [40]. Resurfacing has been suggested as an alternative for younger, active patients, with relatively low revision rates and good function after two to nine years [17, 41–45]. Progress in implant design and technology may have significant influence on revision rates, as metal-on-metal HR is associated with increased requirement for revision only when discontinued devices are included in analyses, as shown in a recent review article [46]. It is yet unclear to what extent surgical technique and implant design affect survivorship, which highlights the requirement for more research related to analysis of benefits and limitations of resurfacing [23]. There have been a number of different studies dealing with comparisons of traditional THA and resurfacing, with varying results related to implant survivorship [46], and they reported similar patient-reported outcomes or outcomes favoring resurfacing [7, 16]. In our study, the outcomes of resurfacing were similar to the neck-preserving MiniHip treatment. Both groups showed significant improvements of all HOOS subscales, but there were no differences between groups or interactions. Considering the younger age of individuals included in this study, both approaches may be equally valuable for this specific group of patients and potentially preferable over traditional implant designs which require a total resection of the femoral neck and require extraction of increased amounts of bone mass (from the femoral component). The bone-sparing characteristics of both interventions are important for recovery after surgery and postsurgery function and in case of potential revisions due to failure or deterioration later in life. Resurfacing may be superior regarding this aspect, since it also does not require significant invasion of the femoral canal. Our results are in tune with several other studies comparing resurfacing and a variety of short-stem implants. In a finite element study, short-stem implants were evaluated as being comparable to resurfacing in terms of bone remodeling, while no bone mineral density loss was reported [47]. Different designs of short-stem implants have been shown to provide good stability, high levels of function, and low revision rates in the short term [6, 48], after two years [49, 50], and after about four to 15 years of follow-up [51–53]. It has been reported that short (or ultrashort) cementless designs are associated with high stability of fixation (while not requiring diaphyseal fixation in addition to metaphyseal fitting), in both older and younger patients [54].

In the current study, the observed results related to HOOS outcomes were supplemented by results from an analysis of surgery duration. The increased complexity and associated higher demands of a HR intervention lead to significantly longer duration of the surgery. This has implications related to costs and efficiency, specifically in view of the similar patient-reported outcomes of both interventions. Despite the challenging nature of HR [39], it could be argued that a lack of experience and associated learning curve may to some extent contribute to the observed results. Additional research should aim at a comparison of methods with similar levels of training for each surgery technique. It is expected that with more experience overall surgery duration will decline, but potentially not to a large extent.

There are several limitations to this study. The matching process conducted to compare two homogenous groups included one major category (age). Despite the fact that initial testing was conducted to determine whether there may be significant demographic differences (e.g., regarding BMI) between both groups, there may be other undetected confounding factors. Another limitation was lack of surgery duration data from a number of patient cases; therefore it may be possible that results are confounded by missing data points.

Both groups consisted of a high percentage of male individuals, which raises the question whether gender may have a significant influence on the outcomes from either treatment. In a separate analysis, we did not find any significant interactions between gender and treatment; however, sample size and associated statistical power for this analysis were small due to the small number of female patients in the study.

Comparison of the MiniHip implant design and resurfacing showed similar patient-reported outcome in the current study. Considering the increased complexity, associated surgery duration, and associated costs, MiniHip surgery may be preferable in younger adults requiring hip arthroplasty. Orthopedic surgeons face the challenge of providing treatments that both are cost-effective and lead to the best possible outcome. It is possible that benefits associated with certain techniques may not suffice to justify the higher cost for certain patient groups [55]. More research is required to further investigate specific advantages or disadvantages. In the current study, the follow-up period was rather short; a planned future follow-up study will shed more light on potential longer-term benefits or limitations. Additionally, considering the potential effects of resurfacing and other less invasive techniques on function in younger, active adults, it will be rewarding and meaningful to analyze results from balance or gait testing. This will allow researchers to draw objective conclusions from observed results.

## Paper Summary

*Article Focus*

- There is no consensus regarding the best treatment for osteoarthritis in younger adults. Here, we compared patient-reported outcome from hip resurfacing versus neck-preserving short-stem total hip arthroplasty.
- We hypothesized that patient-reported outcome would be similar in both treatment groups, while surgery duration would be longer in the hip resurfacing intervention.

*Key Messages*

- There were no differences regarding patient-reported outcome between treatments for adults younger than 56 years of age.
- Surgery duration was significantly longer for hip resurfacing.
- Total hip arthroplasty using a short-stem, neck-preserving implant design may be a valuable, simpler alternative to hip resurfacing.

*Strengths and Limitations of This Study*

- No study yet has compared the modern arthroplasty surgery technique/associated implant design presented here (MiniHip) with hip resurfacing in younger adults.
- The study provides evidence for the value of an alternative to resurfacing in younger adults.
- The number of patients was limited, with an uneven gender distribution.
- There were potential confounding factors (due to matched-cohort study design).
- Missing data points related to surgery duration.

## Figures and Tables

**Table 1:** Demographics of patient groups.

	Gender	Age (in years)	Weight (in kg)	Height (in cm)	BMI
MiniHip	*N* = 73, male = 55	48.2 ± 6.6	91.1 ± 19.2	177.2 ± 8.8	28.8 ± 4.5
	(75.3%), female = 18				

Resurfacing	*N* = 52, male = 47	48.9 ± 6.1	96.6 ± 19.6	181.2 ± 8.6	29.3 ± 5.2
	(90.4%), female = 5				
